# Endoscopic endonasal approach for resection of a recurrent spheno-orbital meningioma resulting in complete resolution of visual symptoms: A case report and review of literature

**DOI:** 10.1007/s11060-022-04141-1

**Published:** 2022-11-29

**Authors:** Won Kim, Farinaz Ghodrati, Khashayar Mozaffari, H. Milan Samarage, Ashley B. Zhang, Anjali Pradhan, Jivianne T. Lee, Robert A. Goldberg, Isaac Yang

**Affiliations:** 1 Department of Neurosurgery, University of California, 300 Stein Plaza, Suite 562, Los Angeles, CA 90095-1761 USA; 2 Radiation Oncology, Los Angeles, CA USA; 3 Head and Neck Surgery, Los Angeles, CA USA; 4 Ophthalmology, Los Angeles, CA USA; 5 Jonsson Comprehensive Cancer Center, Los Angeles, CA USA; 6 Los Angeles Biomedical Research Institute, Los Angeles, CA USA; 7 Harbor- UCLA Medical Center, Los Angeles, CA USA; 8 David Geffen School of Medicine, Los Angeles (UCLA), Los Angeles, CA USA

**Keywords:** Spheno-orbital meningioma, Endonasal, Endoscopic approach, Neurosurgery, Optic tract, Vision loss

## Abstract

**Purpose:**

Spheno-orbital meningiomas are rare tumors, accounting for up to 9% of all intracranial meningiomas. Patients commonly present with proptosis, and visual deficits. These slow growing tumors are hard to resect due to extension into several anatomical compartments, resulting in recurrence rates as high as 35–50%. Although open surgical approaches have been historically used for resection, a handful of endoscopic approaches have been reported in recent years. We aimed to review the literature and describe a case of spheno-orbital meningioma with severe vision loss which was resected with an endoscopic endonasal approach achieving complete resolution of visual symptoms.

**Methods:**

A systematic review of literature was conducted in accordance with the PRISMA guidelines. PubMed, Cochrane, and Web of Science databases were queried for spheno-orbital meningiomas resected via an endoscopic endonasal approach. Furthermore, the presentation, surgical management, and post-operative outcomes of a 53-year-old female with a recurrent spheno-orbital meningioma are described.

**Results:**

The search yielded 26 articles, of which 8 were included, yielding 19 cases. Average age at presentation was 60.5 years (range: 44–82), and 68.4% of patients were female. More than half of the cases achieved subtotal resection. Common complications associated with endoscopic endonasal surgery included CN V2 or CN V2/V3 hypoesthesia. Following surgical intervention, visual acuity and visual field remained stable or improved in the majority of the patients.

**Conclusion:**

Endoscopic approaches are slowly gaining momentum for treatment of spheno-orbital meningiomas. Further studies on the clinical benefits of this approach on patient outcomes and post-operative complications is warranted.

**Supplementary Information:**

The online version contains supplementary material available at 10.1007/s11060-022-04141-1.

## Introduction

Meningiomas are the most common primary brain tumor, accounting for 38.3% of all central nervous system tumors, with an annual incidence rate of 8.81 per 100,000 persons in the U.S. [1]. Originally named “meningioma en plaque” by Cushing and Eisenhardt in 1938, spheno-orbital meningiomas are rare and complex tumors, accounting for up to 9% of all intracranial meningiomas [2–4]. Patients commonly present with symptoms including proptosis, and unilateral deficits in both visual acuity and visual field [3, 5, 6]. Ophthalmoplegia and cognitive problems can also be present in a minority of cases [5].

Spheno-orbital meningiomas are associated with significant hyperostosis of the sphenoid wing and usually extend into the orbital apex anteriorly and the cavernous sinus posteriorly with marked orbital and optic nerve compression [3, 5]. Complete resection of these tumors has been historically challenging given their extensive orbital, bony, and dural involvement, with recurrence rates of 35–50% [3, 6, 7]. A recent meta-analysis found that upwards of 90% of published studies in the literature describe using the pterional or frontotemporal craniotomy approach for resection of spheno-orbital meningiomas [5]. Although recent technological advances have made endoscopic approaches gain momentum, to date only a handful of cases have been presented that use an endoscopic approach to resect these tumors [5]. Here we describe a rare case of spheno-orbital meningioma with severe vision loss resected with an endoscopic endonasal approach achieving complete resolution of visual symptoms. Furthermore, we review the literature on all previously reported cases of spheno-orbotal meningiomas surgically managed via an endoscopic endonasal approach.

## Materials and methods

### Case report

#### Patient consent and IRB approval

All procedures performed in this study involving human participants were in accordance with the ethical standards of the institutional and/or national research committee and with the 1964 Helsinki declaration and its later amendments or comparable ethical standards. For this type of study formal consent or IRB approval was not required.

#### Literature review

**Fig. 1:**
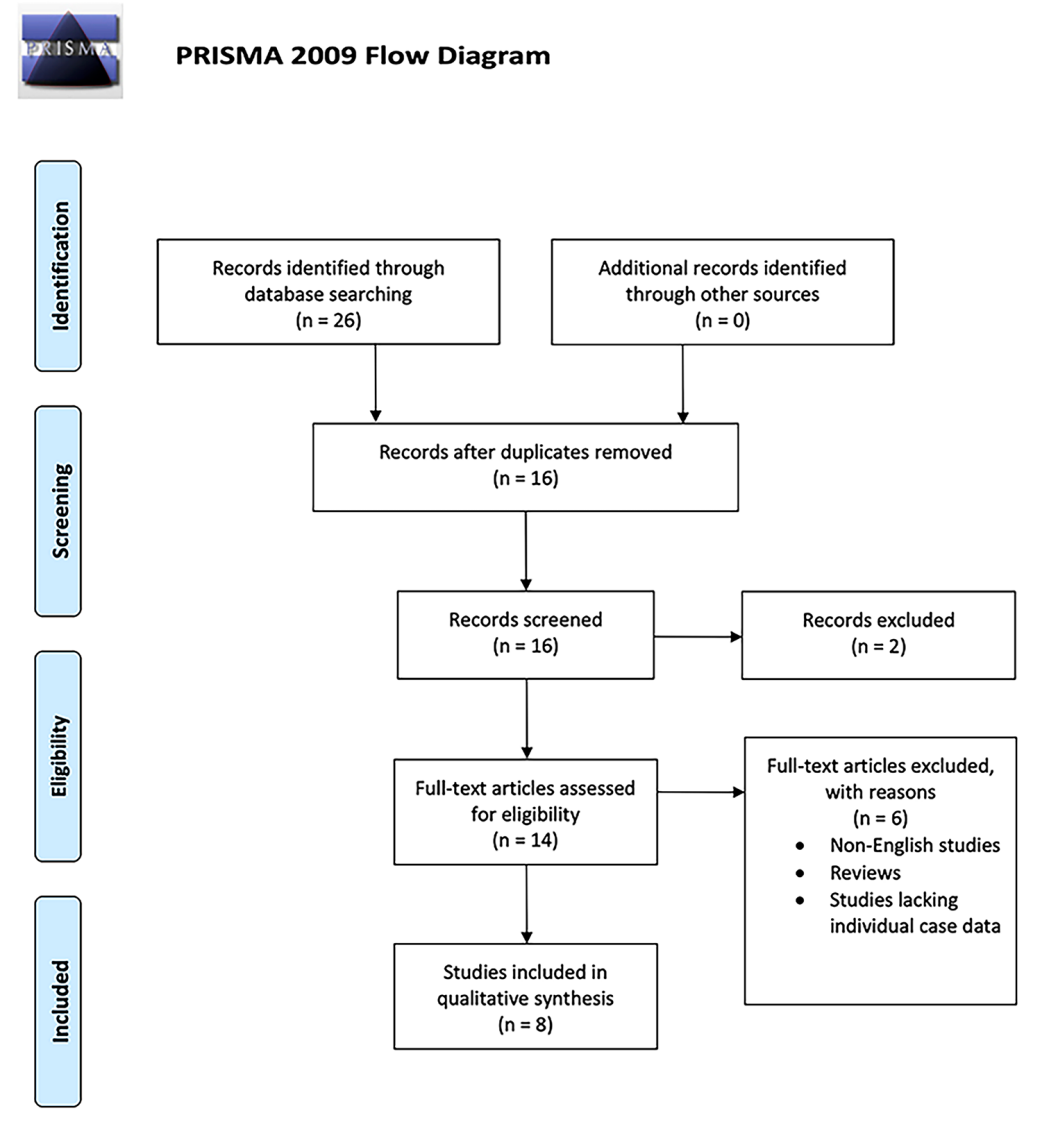
PRISMA flow diagram of the systematic review of the literature on spheno-orbital meningiomas resected via an endoscopic endonasal approach. (From: Moher D, Liberti A, Tetzlaff J, Altman DG, The PRISMA Group (2009). Preffered Reporting Items for Systematic Analysis: The PRISMA Statement. PLos Med 6(6): e1000097. doi: 10.1371/journal.pmed1000097. For more information, visit www.prisma-statement.org.)

A systematic review of literature was conducted in October 2021 in accordance with the Preferred Reporting Items for Systematic Reviews and Meta-analyses (PRISMA) guidelines (Fig. 1**)**. The PubMed, Cochrane, and Web of Science databases were queried using the search terms “sphenoorbital meningioma” or “spheno-orbital meningioma”, and “endonasal”. The search yielded 26 articles, 8 were found to be relevant to this review following exclusion of duplicates, review articles, articles lacking individual case data, and articles on spheno-orbital meningiomas resected using a surgical technique other than endoscopic endonasal approaches. Extracted variables from articles included demographic data, presenting symptoms, tumor characteristics including recurrence status and pathology, surgical management approach, extent of resection, post-operative complications, as well as post-operative status of visual acuity and visual field of patients.

## Results

### Case report

#### Presentation

A 53-year-old female with a history of well-controlled Behçet’s disease presented with decreased visual acuity, visual field defects, and compressive optic neuropathy in the left eye. Surgical history was notable for prior stereotactic left orbitozygomatic craniotomy and subtotal resection (STR) of a World Health Organization (WHO) grade I spheno-orbital meningioma of secretory and microcystic type, five years ago. At the time, the patient presented with afferent pupillary defect, proptosis, and progressively worsening and blurry vision in the left eye, which were all resolved following resection with visual acuity of 20/20 and color vision restored. Mild proptosis recurred a year after and is present to date. The five-year interval was unremarkable other than routine brain imaging and ophthalmology visits until the patient presented with diminished vision in the left eye.

**Fig. 2:**
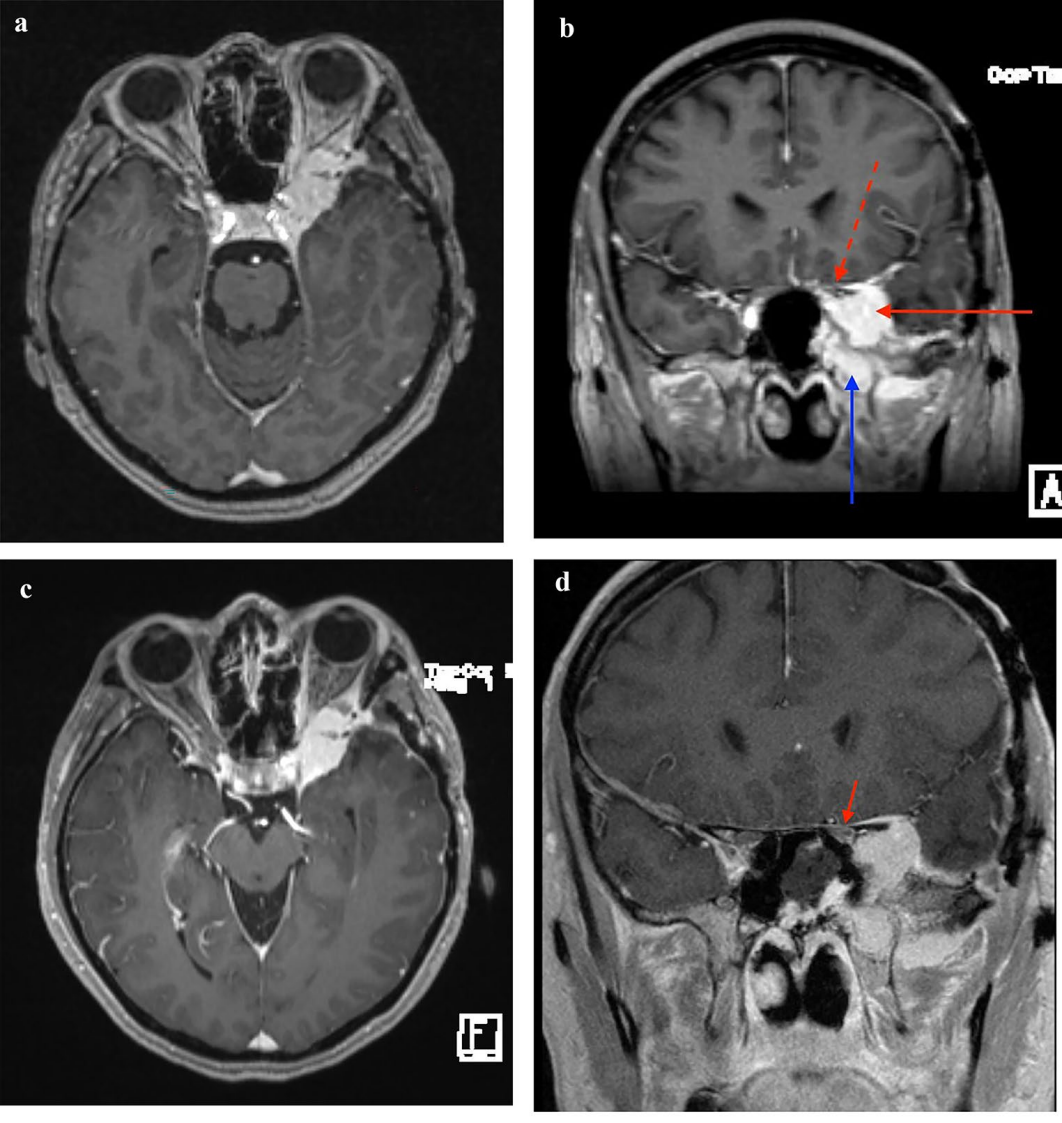
Brain magnetic resonance imaging of the patient. **(A)** Pre-operative axial image demonstrates significant orbital invasion via the superior orbital fissure **(B)** Pre-operative coronal image demonstrating the significant lateral cavernous sinus component of the tumor (solid red arrow), tumor bulk within the pterygopalatine fossa/infraorbital space (solid blue arrow), as well as optic nerve compression (dashed red arrow) **(C)** Post-operative axial image demonstrating no significant debulking of the intraorbital tumor **(D)** Post-operative coronal image 180 degree decompression of the left optic nerve, medial to the tumor bulk (solid red arrow)

Testing showed reduced quality of vision and visual acuity with darkening of vision. Visual field was also defected in the left eye. There was no evidence of recurrent inflammatory disease associated with Behçet’s disease during ophthalmology examinations and laboratory studies. Patient also reported episodes of clear nasal drainage out of the left nostril. Magnetic resonance imaging (MRI) showed interval growth of residual tumor of the left spheno-orbital mass centered in the left middle cranial fossa with extension into the left orbital apex, cavernous sinus, skull base foramina, and masticator space **(**Fig. 2A, 2B**)**. The tumor measured approximately 33 mm TR x 17 mm AP. The patient also had compressive optic neuropathy. Vision continued to decline gradually from 20/24 to 20/100 in the span of 6 months leading up to the resection.

#### Surgical course

**Fig. 3:**
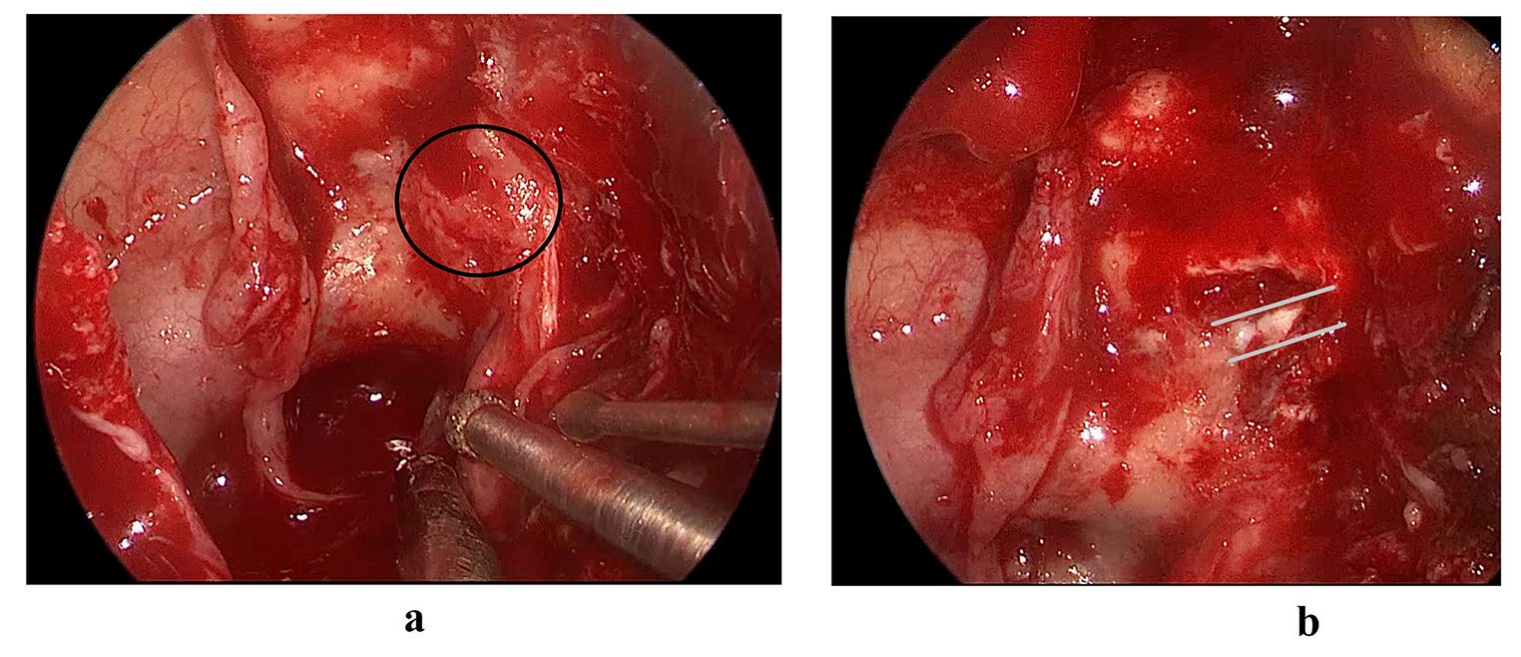
Intraoperative images of the transpterygoid decompression of the optic nerve and orbit. **(A)** Pre-decompression surgery demonstrating hyperostotic bone over the medial opticocarotid recess and optic canal (encircled). **(B)** Post-decompression surgery demonstrating the dura of the optic canal (grey lines), freed of the bony and tumoral compression

The patient underwent resection of the tumor using an endoscopic endonasal transpterygoid approach, and endoscopic optic nerve decompression (**Supplementary Video)**. Following the raising of the nasoseptal flap on the patient’s right side, a left transpterygoid approach was performed to access the pterygopalatine fossa, filled by the tumor. With a 2-surgeon bimanual approach and binostril technique, STR of the tumor was achieved **(**Fig. 2C, 2D**)**. The lamina papyracea was removed from the medial orbital wall, and the periorbital opened sharply to allow for orbital decompression (Fig. 3A, 3B**)**. The nasoseptal flap was used for skull base reconstruction of the middle cranial fossa given the history of clear rhinorrhea, suspicious for cerebrospinal fluid leak [8, 9, 10]. There were no complications associated with the surgery. The pathology report found a WHO grade I meningioma of secretory type.

#### Post-operative course

The patient’s vision gradually improved, and a visual acuity test in last follow-up showed 20/20 vision. Preoperative visual field deficits in the left eye recovered. The patient reported new-onset cranial nerve (CN) V2 hypoesthesia over maxilla and hard palate following surgery, which has also gradually ameliorated since surgery. Her sense of taste and smell was diminished post-operatively but has since improved as reported in the last follow-up visit. The patient underwent fully fractionated intensity modulated radiation therapy (IMRT) to 50.4 Gy for the residual tumor, with no evidence of recurrence to date.

## Literature review

Our review of literature yielded 8 articles published between 2013 and 2020 on 19 cases of spheno-orbital meningiomas resected via an endoscopic endonasal approach. Patient demographics and case characteristics are listed in Tables 1 and 2, respectively. 68.4% of patients were female and the average presenting age was 60.5 years (range: 44–82). 63.1% of patients had WHO grade I, 5.3% WHO grade II, 5.3% WHO grade III meningiomas, and the remainder of cases did not report WHO grading. 52.6% of cases comprised of recurrent meningiomas, 36.8% were primary tumors while the rest of the cases did not report on recurrence. The most common presenting symptoms were proptosis (57.9%), visual impairment (47.4%), optic neuropathy (15.8%), and CN V2/V3 hypoesthesia (10.6%). Extraoccular movement limitation, painless swelling of the left orbitotemporal region, CN III and VI nerve deficit, and midfacial numbness each presented at 5.3% amongst the patients.

**Table 1:** Patient demographics of spheno-orbital meningiomas surgically treated with an endoscopic endonasal approach in the literature

		Patients (n = 19)
**Sex**		
	Female	13 (68.4%)
	Male	6 (31.6%)
**Age**		
	Mean	60.5
	Median	61
	Range	44–82
**Presenting Symptoms**		
	Visual impairment	9 (47.4%)
	Proptosis	11 (57.9%)
	CN V2/V3 hypoesthesia	2 (10.6%)
	Optic neuropathy	3 (15.8%)
	Extraocular movement limitation	1 (5.3%)
	Painless swelling of the left orbitotemporal region	1 (5.3%)
	CN III and VI nerve deficit	1 (5.3%)
	Midfacial numbness	1 (5.3%)
**WHO Grade**		
	I	12 (63.1%)
	II	1 (5.3%)
	III	1 (5.3%)
	NR	5 (26.3%)
**Recurrent**		
	Yes	10 (52.6%)
	No	7 (36.8%)
	NR	2 (10.6%)
**Surgical Approach**		
	Combined EEA and FT craniotomy	6 (31.6%)
	Combined EEA and ETA	6 (31.6%)
	EEA	1 (5.3%)
	EEA, secondary FT craniotomy	3 (15.8%)
	FT craniotomy, secondary EEA	2 (10.6%)
	Combined EEA and transzygomatic craniotomy	1 (5.3%)
**Extent of resection**		
	STR	11 (57.9%)
	GTR	3 (15.8%)
	NR	5 (26.3%)
**Endoscopic Endonasal Surgery Complications**		
	CN V2 hypoesthesia	2 (10.6%)
	CN V2/V3 hypoesthesia	1 (5.3%)
	None	9 (47.4%)
	NR	7 (36.8%)
**Visual Acuity Status Post-op**		
	Improved	6 (31.6%)
	Stable	3 (15.8%)
	Stable deficit	2 (10.6%)
	Worsened	2 (10.6%)
	NR	6 (31.6%)
**Visual Field Status Post-op**		
	Improved	3 (15.8%)
	Stable	4 (21.1%)
	Stable concentric deficit	1 (5.3%)
	Stable temporal deficit	1 (5.3%)
	Worsened	1 (5.3%)
	NR	9 (47.4%)

CN = cranial nerve; WHO = World Health Organization; NR = not reported; EEA = endoscopic endonasal approach; FT = frontotemporal; ETA = endoscopic transorbital approach; STR = subtotal resection; GTR = gross total resection; post-op = post-operatively

**Table 2:** Review of literature on spheno-orbital meningiomas surgically treated with an endoscopic endonasal approach

Case No.	Author (year)	Age	Sex	Presenting symptoms	Surgical approach	Extent of resection	Endoscopic endonasal surgery complications	WHO Grade	Recurrent	Visual acuity status post-op	Visual field status post-op
1	Present study (2021)	53	F	Visual impairment, proptosis,	Endoscopic endonasal transpterygoid approach	STR	CN V2 numbness (resolved by last follow-up), diminished sense of taste and smell (improved by last follow-up)	Grade I	X	Improved	Improved
2	Matsuda et al. (2020)^16^	62	M	Proptosis	Combined frontotemporal craniotomy and endoscopic endonasal transsphenoidal plus transmaxillary transpterygoid approach	STR	None	Grade I	X	NR	NR
3	Matsuda et al. (2020)^16^	70	M	Proptosis	Combined frontotemporal craniotomy and endoscopic endonasal transsphenoidal plus transmaxillary transpterygoid approach	STR	CN V2 numbness	Grade I	X	NR	NR
4	Matsuda et al. (2020)^16^	63	F	CN V2/3 numbness	Combined frontotemporal craniotomy and endoscopic endonasal transsphenoidal plus transmaxillary transpterygoid approach	GTR	CN V2/V3 hypoesthesia	Grade I	X	NR	NR
5	Matsuda et al. (2020)^16^	70	F	CN V2/3 numbness	Combined frontotemporal craniotomy and endoscopic endonasal transsphenoidal plus transmaxillary transpterygoid approach	STR	CN V2 hypoesthesia	Grade I		NR	NR
6	Kong et al. (2019)^8^	51	F	Optic neuropathy, proptosis, and extraocular movement limitation	Combined endoscopic transorbital and endonasal approach	STR	NR	Grade I	X	Stable deficit	NR
7	Kong et al. (2019)^8^	52	F	Optic neuropathy, and proptosis	Combined endoscopic transorbital and endonasal approach	STR	NR	Grade I	X	Stable deficit	NR
8	Kong et al. (2019)^8^	60	F	Optic neuropathy, visual impairment	Combined endoscopic transorbital and endonasal approach	GTR	NR	Grade I		Improved	NR
9	Almeida et al. (2018)^9^	53	F	Visual impairment, and proptosis	Combined endoscopic transorbital and endonasal approach	STR	None	Grade I	NR	Stable	Improved
10	Almeida et al. (2018)^9^	65	F	Proptosis, facial numbness, visual impairment	Combined endoscopic transorbital and endonasal approach	STR	None	Grade I	NR	Improved	Stable
11	Corniola et al. (2017)^18^	74	F	Proptosis, painless swelling of the left orbitotemporal region	Frontotemporal craniotomy, secondary endoscopic endonasal technique via medial maxillectomy	NR	None	Grade III		NR	NR
12	Matsuda et al. (2017)^10^	62	M	Proptosis	Combined transzygomatic and endoscopic endonasal approaches	STR	None	Grade I	X	Stable	Stable
13	Dallan et al. (2015)^12^	67	M	Proptosis, and midfacial numbness	Combined endoscopic multiportal transnasal-transorbital approach	STR	None	NR	X	Stable	Stable
14	Berhouma et al. (2014)^17^	54	F	Visual impairment	Endoscopic endonasal optic nerve decompression, secondary frontotemporal craniotomy	NR	NR	NR		Improved	Stable
15	Berhouma et al. (2014)^17^	61	M	Visual impairment	Endoscopic endonasal optic nerve decompression, secondary frontotemporal craniotomy	NR	NR	NR		Worsened	Stable temporal deficit
16	Berhouma et al. (2014)^17^	56	F	Visual impairment	Endoscopic endonasal optic nerve decompression, secondary frontotemporal craniotomy	NR	NR	NR		Improved	Stable concentric deficit
17	Berhouma et al. (2014)^17^	49	F	Visual impairment	Endoscopic endonasal optic nerve decompression	NR	NR	NR		Improved	Improved
18	Attia et al. (2013)^4^	55	M	Proptosis	Frontotemporal Craniotomy, secondary endoscopic endonasal transsphenoidal/transethmoidal/ transpterygoid approach	STR	None	Grade I	X	Worsened	Worsened
19	Attia et al. (2013)^4^	82	F	Visual impairment, and proptosis	Combined Frontotemporal craniotomy and endoscopic endonasal transsphenoidal/transethmoidal approach	GTR	None	Grade I	X	NR	NR
20	Attia et al. (2013)^4^	44	F	Visual impairment, and CN III and VI nerve deficit	Combined Frontotemporal craniotomy and endoscopic endonasal transsphenoidal/transethmoidal/ transpterygoid approach	STR	None	Grade II	X	Improved	Improved

No. = number; WHO = World Health Organization; F = female; M = male; X = Yes; CN = cranial nerve; STR = subtotal resection; NR = not reported; GTR = gross total resection; Blank space = No

In all but 1 of the cases, a combined approach was used for the resection of the tumor, which constituted of both simultaneous or secondary surgeries. The combined endoscopic endonasal approach and frontotemporal craniotomy was used in 31.6% of patients, combined endoscopic endonasal approach and transorbital approach in 31.6%, solely endoscopic endonasal approach in 5.3%, endoscopic endonasal approach with a secondary frontotempotal craniotomy in 15.8%, frontotemporal craniotomy with secondary endoscopic endonasal approach in 10.6%, and combined endoscopic endonasal and transzygomatic approach in 5.3% of cases. Gross total resection (GTR) was achieved in 15.8% of patients while 57.9% had STR, and extent of resection was not reported in the remaining patients.

The complications associated with endoscopic endonasal surgery included CN V2 hypoesthesia (10.6%), CN V2/V3 hypoesthesia (5.3%), none (47.4%), and not reported in the remainder of the cases. Post-operative visual acuity status was improved in 31.6% of cases, stable in 15.8%, stable deficit in 10.6%, worsened in 10.6%, and not reported in the rest of the cases. Post-operative visual field status was improved in 15.8% of patients, stable in 21.1%, stable deficit in 10.6%, worsened in 5.3%, and not reported for the remaining cases.

## Discussion

Spheno-orbital meningiomas are rare tumors that commonly present with visual impairment and proptosis [5, 11]. Although these are usually slow-growing tumors, their considerable involvement with bony, dural, and orbital structures renders their safe GTR challenging [3, 4, 6, 7, 11–13]. The need for significant resection of sphenoid and orbital bones further limits resection in these tumors, as it can lead to cranial deformity resulting in post-operative complications, morbidity, and mortality [3, 12]. Given that complete resection is often unattainable, residual growth and recurrence is common, with adjuvant radiotherapy and reoperation often required in management of these tumors [3, 4, 12]. Although historically open approaches, such as frontotemporal craniotomy, were used for the resection of these tumors, in recent years, minimally invasive approaches have become available [12, 14]. Open approaches provide a wider exposure to tumor tissue; however, they may be associated with functional and cosmetic post-operative complications, which can be avoided in minimally invasive techniques [12, 15]. To date, there remains no consensus surrounding the reconstruction of orbital walls following tumor resection, with proponents of bony construction deeming it a necessity to reduce the occurrence pulsatile exophthalmos [16]. A recent meta-analysis found that the pterional approach relieves visual symptoms including diplopia, ophthalmoplegia, and visual acuity and field deficits in approximately 90% of patients [5]. However, post-operative complications are commonly present with approximately 20% post-operative occurrence of hypoesthesia, ophthalmoplegia, diplopia, and ptosis in patients [5].

Minimally invasive endoscopic endonasal approaches have changed the paradigm when it comes to the management of midline skull base tumors and in select cases have been able to achieve more favorable resection and complication rates, compared to open approaches [12, 14]. Our literature review of the 19 cases of endonasal endoscopic approaches showed no post-operative complications in 75% of patients and CN V2/V3 hypoesthesia in the remaining 25%. Here we described a case of an endoscopic endonasal transpterygoid approach for surgical debulking of a spheno-orbital meningioma and optic nerve decompression resulting in complete resolution of the patient’s visual acuity and visual fields. Classically, endoscopic endonasal procedures are favored in tumors that are predominantly medial to the optic canal and carotid artery given the limitations of this technique. However, despite the significant component of the tumor lateral to the optic nerve within the cavernous sinus, this approach allowed for the bony and tumoral decompression of the optic nerve and orbit, without the morbidity of diplopia often associated with debulking tumors within the cavernous sinus and orbit. Resolution of visual symptoms was achieved despite significant residual within the cavernous sinus (Fig. 2C**)**. Following resection, the patient reported CN V2 hypoesthesia and diminished sense of taste and smell, which were both improved in a follow-up visit four months post-operatively. The endoscopic endonasal approach has been found beneficial for optic nerve decompression as it provides excellent exposure of the medial orbital apex and the optic canal [14, 17].

Other endoscopic methods, such as the transorbital approach have also been utilized for tumors located laterally [12, 15]. The surgical approach is indeed dependent on tumor location with the endoscopic endonasal technique affording great visualization of orbital apex and optic canal and allowing resection of tumors extended to the medial optic canal, pterygopalatine fossa and the infratemporal fossa [12, 14, 15, 17]. In contrast transorbital endoscopic approaches provide good exposure of the lateral wall and orbital roof [12, 15]. It should be noted that endoscopic approaches for resection of spheno-orbital meningiomas were described fairly recently and have been used in only a handful of studies with the earliest article in our literature review published in 2013 [4]. More studies are warranted to further evaluate this approach to improve patient outcomes and post-operative complications with the goal of improving quality of life.

## Conclusions

Although open surgical approaches such as frontotemporal craniotomy were the gold standard of treatment for spheno-orbital meningiomas, endoscopic approaches are slowly gaining momentum. Here we presented a successful case of spheno-orbital meningioma resection with an endoscopic endonasal approach with good outcomes. More studies are needed on the utility of endoscopic endonasal approaches for spheno-orbital meningiomas. However, in select patients, this may serve as a favorable, minimally invasive alternative with low morbidity in patients where complete tumor resection is not possible.

## Electronic supplementary material

Below is the link to the electronic supplementary material.

Supplementary Material 1
